# Adult Low‐Grade Glioma With *MEF2D::NTRK1* Fusion: A Case Report and Literature Review

**DOI:** 10.1155/crip/6015225

**Published:** 2026-10-07

**Authors:** Xiuyi Pan, Linmao Zheng, Xiaoxue Yin, Mengxin Zhang, Ting Yu, Zhengzheng Su, Xueqin Chen, Ni Chen, Yuyan Wei

**Affiliations:** ^1^ Department of Pathology and State Key Laboratory of Biotherapy West China Hospital Sichuan University Chengdu China scu.edu.cn

**Keywords:** diagnosis, DNA methylation, low-grade glioma, *MEF2D::NTRK1*, targeted therapy

## Abstract

Low‐grade glioma (LGG) is a malignancy that is rare in adult central nervous system (CNS). Clinical management decisions are increasingly guided by molecular alterations rather than the histological subtype alone. Neurotrophic tropomyosin receptor kinase 1 (*NTRK1*) is constitutively activated in multiple tumor types. TRK inhibitors, targeting NTRK fusion proteins, have exhibited notable therapeutic efficacy in some *NTRK* gene fusion tumors. NTRK fusions with diverse partners have been reported in CNS tumors. Nevertheless, *MEF2D::NTRK1* fusion cases were only reported in high‐grade glioneuronal neoplasms and infantile‐type hemispheric glioma. We report a 33‐year‐old patient with LGG morphological features, but presented a diffuse invasive growth pattern involving the corpus callosum, the third ventricle, the entire anterior cranial base and the suprasellar cistern. Histology showed oligodendrocyte‐like cells within an abundant mucinous matrix, with scattered astrocytes and no dense collagen, Rosenthal fibers or glomeruloid microvascular. The nucleoli were inconspicuous and mitotic figure was rare. Next‐generation sequencing (NGS) showed it harbored a *MEF2D::NTRK1* fusion and 22q loss with no additional mutations identified. DNA methylation clustered with pilocytic astrocytoma (PA) and diffuse leptomeningeal glioneuronal tumor (DLGNT). However, according to the tumor′s growth pattern, morphological features, and molecular characteristics, it does not match to PA or DLGNS. This report provides a comprehensive description of the clinical, radiographic, histopathological, immunohistochemical, and molecular characteristics of *MEF2D::NTRK1* fused LGG with the aim of enhancing the clinical and pathological understanding of *MEF2D::NTRK1* fused LGG. Patient in this case had benefited from *NTRK*‐targeted therapeutic intervention.

## 1. Introduction

Gliomas are brain tumors characterized by intratumoral heterogeneity. The diagnosis of these tumors is based on their histological features and distinct molecular alterations, as outlined in the 2021 World Health Organization (WHO) classification of central nervous system (CNS) tumors [1]. The fifth edition of the WHO CNS tumor classification system mainly distinguishes gliomas in children and adults, and subsequently classifies them as diffuse or localized on the basis of growth pattern and infiltrative behavior [1].

LGGs are most often diagnosed in children and young adults and concern Grades I and II tumors. The Grade I tumor is the pilocytic astrocytoma (PA) and Grade II tumor includes oligodendroglioma and Grade II diffuse astrocytoma [2]. Although LGGs exhibit a slow growth feature compared with their malignant counterparts, they still have the potential to undergo malignant transformation into high‐grade gliomas, which is associated with a poor prognosis. LGGs often show an infiltrative growth pattern, a characteristic that inherently precludes the possibility of achieving a surgical cure. Therefore, there exists no universally accepted or standardized treatment protocol for this condition. Adult LGGs are more treatment‐resistant than those in children. In LGGs, the most frequent molecular alterations include 1p/19q codeletion, mutations in isocitrate dehydrogenase 1/2 (*IDH1*/*2*) and *TP53*, O‐6‐methylguanine‐DNA methyltransferase (*MGMT*) promoter methylation [3].

Gene fusions can produce novel transcripts that encode aberrant functional proteins, a phenomenon well‐documented in glioma [4, 5]. The neurotrophic tropomyosin receptor kinase (NTRK) family consists of three transmembrane tyrosine kinases—NTRK1, NTRK2, and NTRK3—each containing an extracellular ligand‐binding domain. These receptors function as oncogenes and mediate prosurvival signaling in response to nerve growth factor. In various tumor types, NTRKs can be constitutively activated through fusion events. This frequently eliminates the ligand‐binding domain, leading to ligand dimerization and phosphorylation of TRK receptors, which in turn results in constitutive activation of downstream signaling pathways [6]. Targeted inhibitors against TRK receptors, such as larotrectinib, have been shown to effectively counteract disease progression in tumors harboring *NTRK* fusions [7, 8]. *NTRK* fusions involve a range of interchromosomal partner genes, including *ETV6* [9], *ARHGEF2*, *NFASC*, *BCAN* [10], and *MEF2D* [11]. To date, case reports of *MEF2D::NTRK1* fusion have been associated exclusively with high‐grade glioneuronal neoplasms [12] and infantile‐type hemispheric gliomas [13].

According to the literature, *NTRK* gene fusion can be observed in approximately 10% of infants with high‐grade glioma [14], but occurs at a much lower frequency in pediatric patients with LGG [15–17]. One study identified *TPM3::NTRK1* and *ETV6::NTRK3* fusions as the most common genetic alterations in infant nonbrainstem high‐grade gliomas [18]. In adults, *NTRK* fusions are relatively rare and are primarily reported in high‐grade gliomas and glioblastomas [19–22]. Among these genes, *NTRK2* is the most frequently altered gene in adult glioma [17, 19, 22]. Regarding LGGs, a case of *BCAN::NTRK1* fusion has been reported in an adult PA [19]. These findings highlight the potential of *NTRK* fusions as actionable therapeutic targets in affected patients.

Herein, we report an adult case of LGG harboring a *MEF2D::NTRK1* fusion, *MCL1* amplification, chromosome 5 gain and 22q loss without evidence of *IDH1/2* mutation, 1p/19q codeletion, or *TERT* promoter mutation. Based on the fifth edition of the WHO Classification of CNS tumors, this glioma does not correspond to a well‐defined histological subtype. It has been diagnosed as a low‐grade glioma with a *MEF2D::NTRK1* fusion. To our knowledge, this represents the first documented occurrence of the *MEF2D::NTRK1* fusion in a LGG.

## 2. Case Presentation

### 2.1. Clinical History

A 33‐year‐old male patient was admitted to our hospital after an intracranial mass was incidentally discovered by routine cranial computed tomography (CT) during the patient′s regular health examination. The patient had no subjective neurological or systemic symptoms, including no headache, dizziness, nausea, vomiting, visual impairment, limb weakness, or seizure. Further, magnetic resonance imaging (MRI) revealed a solid mass measuring 5.2 × 4.3 × 2.6 cm located in the midline intracranial region, involving the suprasellar area, bilateral hypothalamus, anteroinferior third ventricle, and inferior corpus callosum (Figure 1A–D). Preoperative diffusion‐weighted imaging (DWI) showed tumor displayed slightly high signal (Figure 1E). In the absence of surgical contraindications, the patient underwent partial resection of the intracranial mass involving the left anterior and middle cranial base. During the surgical procedure, it was observed that the tumor located in the corpus callosum, the third ventricle, the entire anterior cranial base, and the suprasellar cistern, with indistinct demarcation from the adjacent tissue. The tumor exhibited adherence to the lateral fissure vessels as well as the branches of the anterior and middle cerebral arteries. Within 2 weeks after surgery, the patient experienced moderate cognitive and memory decline and was readmitted for the placement of a ventriculoperitoneal shunt. Postoperation MRI was used to evaluate the status of tumor surgical removal, and it revealed slight hemorrhage and cerebral edema in the midline region (Figure 1F–H). His symptoms improved following the shunt procedure.

**Figure 1:**
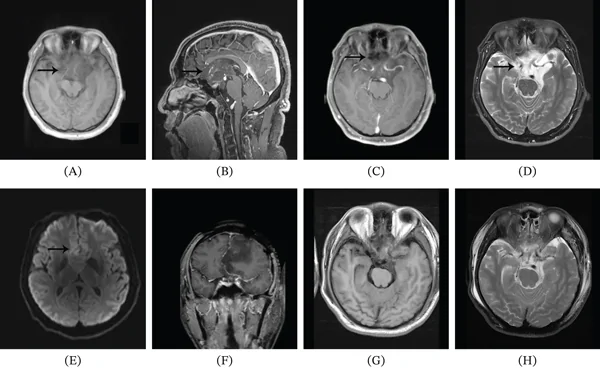
MRI characteristics. (A–E) Preoperative imaging. (A) T1‐weighted image demonstrated a solid, hypointense mass in the intracranial midline region. (B,C) Postcontrast T1‐weighted images (B) sagittal view and (C) axial view showed lesions with low signal intensity. (D) Postcontrast T2‐weighted image revealed an irregular, ill‐defined hyperintense mass in the same region. (E) Preoperation DWI showed that tumor presented slightly high signal. (F,G) Postoperative imaging. (F) Postoperative coronal and (G) axial T1‐weighted scans. (H) Postoperative axial T2‐weighted scan.

### 2.2. Pathological Findings

This intracranial midline tumor presents typical low‐grade tumor morphological characteristics. Grossly, it manifests as a jelly‐like soft mass with diffuse infiltrative growth and unclear boundary with adjacent normal brain tissues, involving multiple critical intracranial structures including the corpus callosum, third ventricle, anterior cranial base, and suprasellar cistern. Microscopically, the tumor is predominantly composed of oligodendrocyte‐like cells arranged in solid sheets, with abundant diffuse mucinous matrix forming a loose cell‐suspended architectural pattern (Figure 2A–D). Focal fibrillary stroma and scattered reactive astrocytes can be observed. The tumor cells show oval nuclei with inconspicuous nucleoli, extremely low mitotic activity, and no typical malignant morphological features including necrosis, glomeruloid microvascular proliferation, dense collagen deposition, and Rosenthal fibers. Uniform arborizing capillaries are diffusely distributed in the lesion, without atypical vascular hyperplasia.

**Figure 2:**
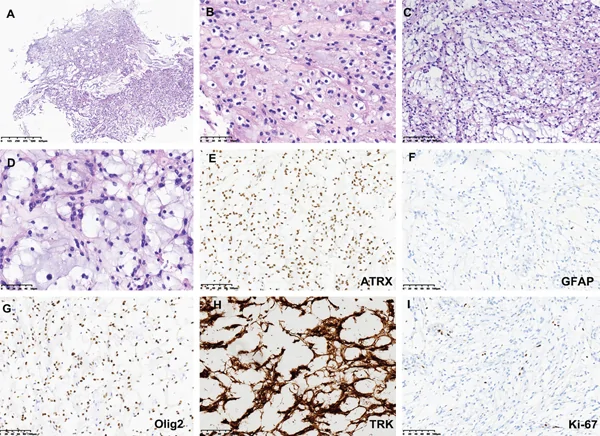
Pathological characterization. (A) The tumor cells were arranged in solid sheets within a prominent mucinous matrix. (B) The tumor cells were predominantly composed of oligodendrocyte‐like cells. (C) A loose architecture was evident in areas rich in mucinous background. (D) Tumor cells exhibited uniform nuclei and rare mitoses activity. (E–I) Immunohistochemical staining showed: (E) retained nuclear expression of ATRX; (F) negativity for GFAP; (G) diffuse nuclear positivity for Olig2; (H) strong cytoplasmic positivity for pan‐TRK; and (I) a low Ki‐67 proliferation index (1%–2%).

Immunohistochemically, the tumor exhibits a mixed glial and glioneuronal immunophenotype. It is diffusely positive for glial lineage markers (Olig2 and ATRX) and stem cell marker SOX2, TRK, as well as neuronal marker Syn, confirming its glioneuronal differentiation origin. Meanwhile, the tumor is negative for GFAP (mature astrocytic marker), IDH1, H3K27M, TTF‐1, and CD34 (Figure 2E–I). The Ki‐67 proliferation index is extremely low (1%–2%), indicating indolent biological behavior, and only focal P53 expression is detected without diffuse overexpression. Epigenetically, the tumor retains H3K27Me3 expression, excluding H3K27M‐mutant diffuse midline glioma.

Molecular and methylation characteristics: Tumor molecular profiling was performed using a commercial targeted next‐generation sequencing (NGS) panel, SimcereDx NeuroOnco360 (Nanjing, China). This panel covers full‐length exons and partial introns of common glioma‐related genes to detect gene mutations, fusions, and copy number variations. Genomic DNA was extracted from FFPE tumor tissue; library construction, target capture, and high‐throughput sequencing were performed following the manufacturer′s standard protocols. NGS testing identified a rare *MEF2D::NTRK1* fusion with breakpoints at *MEF2D* exon 9 and *NTRK1* exon 12. No 1p/19q codeletion was detected (Figure 3A). PCR assays revealed no hotspot mutations in *IDH1*, *IDH2*, or the *TERT* promoter, no *BRAF* fusions, and no *MGMT* promoter hypermethylation. To further validate the NGS‐identified fusion transcript, reverse transcription polymerase chain reaction (RT‐PCR) and Sanger sequencing were conducted (Figure 3C). The primer sequences for *MEF2D::NTRK1* fusion detection were listed as follows: forward primer (FP): TCTCCCAGTCTACTCATTCGC; reverse primer (RP): GAAGTATTGTGGGTTCTCGATG.

**Figure 3:**
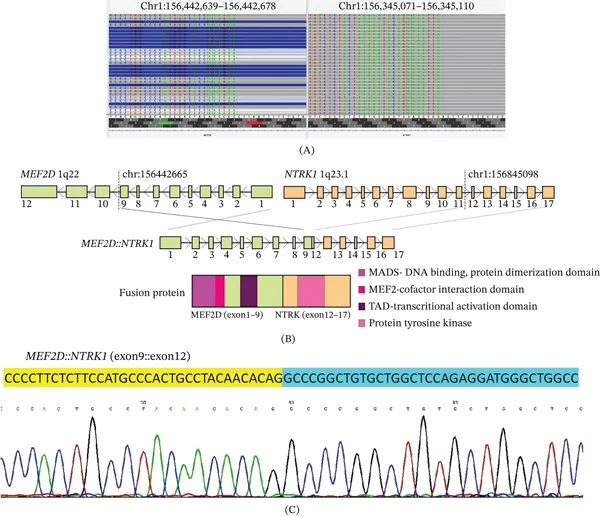
Detection of the *MEF2D::NTRK1* fusion. (A) DNA‐level IGV image of NGS indicating *MEF2D::NTRK1* fusion. (B) Schematic presentation of *MEF2D::NTRK1* fusion, which joins exons 1–9 of *MEF2D* to exons 12–17 of *NTRK1*, and the resulting fusion protein domains. (C) Sanger sequencing chromatogram confirming the fusion junction between *MEF2D* exon 9 and *NTRK1* exon 12.

Furthermore, genome‐wide DNA methylation profiling of CNS tumors was performed by Guangzhou Huayin Medical Laboratory Center using the Infinium MethylationEPIC v2.0 (935K) methylation array. The methylation data were analyzed with a machine learning classifier based on a 2801‐sample training cohort. Chromosomal copy number variations include *MCL1* amplification, chromosome 5 gain, and 22q loss (Figure 4A). The t‐distributed stochastic neighbor embedding (t‐SNE) result showed this case was positioned close to PA (Figure 4B), random forest clustering shows it is categorized as diffuse leptomeningeal glioneuronal tumor. Additionally, we performed an analysis employing the National Institutes of Health (NIH) database, and the findings indicated its classification as low‐grade‐glial‐glioneuronal‐tumors.

**Figure 4:**
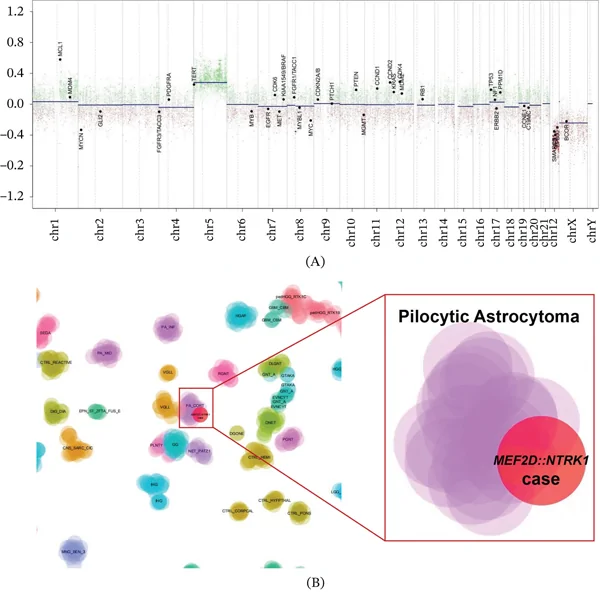
DNA methylation profiling analysis. (A) Copy number variation profile. It revealed *MCL1* amplification, chromosome 5 gain and chromosome 22q loss in this case. (B) t‐SNE plot of DNA methylation‐based classification of central nervous system tumors. Each dot represents a reference tumor sample, color‐coded according to its methylation class. The red‐labeled sample corresponds to the *MEF2D::NTRK1* fusion positive case, which clusters closely with the PA methylation class.

### 2.3. Follow‐Up

The patient declined conventional chemotherapy and initiated targeted therapy with the NTRK inhibitor entrectinib after the resolution of encephaledema and the recovery of cognitive and memory functions. Regular follow‐up imaging examinations were performed externally at a local hospital during targeted treatment, and detailed serial imaging data and original scan images were not available for systematic radiological evaluation. According to the patient′s clinical follow‐up records up to June 2026, he remained clinically stable without neurological deterioration, tumor‐related symptoms, or treatment discontinuation. Given the lack of complete serial postoperative imaging data, systematic radiological assessment of treatment response and formal calculation of progression‐free survival (PFS) could not be performed. Overall survival (OS) was defined as the time from diagnosis to either death or the last follow‐up. The patient has granted written informed consent for the dissemination of associated data and images. All methodological processes were conducted in strict compliance with pertinent guidelines and regulatory requirements.

## 3. Discussion

The fifth edition of the WHO Classification of CNS tumors stratifies gliomas into adult‐ and pediatric‐type entities, with further subdivision into diffuse or localized subtypes based on histological growth pattern and invasive capacity [1]. The classification emphasizes molecularly defined clinical and biological characteristics over a rigid age‐based distinction [23]. Intriguingly, pediatric‐type gliomas may occasionally occur in adult patients, whereas adult‐type gliomas can also be observed in adolescents. This flexible diagnostic framework is critical for classifying rare atypical gliomas with mixed phenotypic features, such as the present case.

Adult pilocytic astrocytomas (APAs) are significantly rarer than pediatric PAs and exhibit a more aggressive clinical prognosis. Classic PAs typically arise in the cerebellum and periventricular regions, presenting as well‐circumscribed, slowly growing localized masses. Histologically, PAs are characterized by solid architecture composed of oligodendrocyte‐like cells with typical “fried egg” morphology, accompanied by pathognomonic Rosenthal fibers and eosinophilic granular bodies. Genetically, APAs are predominantly driven by MAPK pathway aberrations, including *BRAF* fusions, *NF1* loss, and *FGFR1* mutations, frequently accompanied by *CDKN2A/B* deletions. In general, APAs lack *TP53* mutations, *MGMT* promoter methylation, 1p/19q loss of heterozygosity, and *IDH* hotspot mutations [24, 25].

DLGNT is very rare and often occurs in children, rarely found in adults. Because of its rarity, the etiology, progression, and outcomes are limited. The most characteristic feature of DLGNT is that the tumor shows a diffuse leptomeningeal abnormality and composed of oligodendrocyte‐like cells [26]. Typical DLGNT features diffuse leptomeningeal infiltration and monotonous oligodendrocyte‐like cells, occasionally accompanied by Rosenthal fibers and glomeruloid microvascular proliferation [27, 28]. Genetically, DLGNT is often associated with two alterations including chromosomes 1p/19q codeletion and *BRAF* fusion [29].

The present tumor shows partial morphological and epigenetic overlap with both PA and DLGNT, which may cause diagnostic confusion. Morphologically, it consists predominantly of monotonous oligodendrocyte‐like cells, mimicking the cytological features of PA and DLGNT. In addition, DNA methylation profiling confirmed a methylomic signature closely adjacent to these two low‐grade tumors, further leading to phenotypic similarity.

However, multiple key histological, radiological, and molecular distinctions exclude PA and DLGNT. Histopathologically, this tumor exhibits extensive mucinous matrix deposition and a loose architecture, which are not typical of conventional PA and DLGNT. Notably, pathognomonic Rosenthal fibers, eosinophilic granular bodies, and glomeruloid microvascular proliferation were entirely absent. Radiologically, unlike the well‐demarcated PA and leptomeningeal‐dominant DLGNT, this lesion showed a diffuse infiltrative pattern involving the corpus callosum, third ventricle, and anterior cranial base.

Most importantly, molecular profiles are fundamentally different. This case lacks hallmark alterations of PA and DLGNT, including *BRAF* fusions, 1p/19q codeletion, and *IDH* mutations. Instead, it harbors unique alterations, including *MEF2D::NTRK1* fusion, chromosome 22q loss, and *MCL1* amplification. Immunohistochemically, the tumor was positive for Olig2, ATRX, and H3K27Me3 but negative for GFAP and H3K27M, with low proliferative activity, presenting an atypical immunophenotype inconsistent with canonical PA and DLGNT.

In summary, although the oligodendrocyte‐like morphology and methylation profile of this tumor partially resemble PA and DLGNT, its distinctive mucin‐rich loose stroma, diffuse infiltrative growth pattern, and unique NTRK‐fusion–driven molecular features do not fully match the diagnostic criteria of either entity. Based on integrated radiological, pathological, immunohistochemical, and molecular findings in accordance with the 2021 fifth WHO CNS tumor classification, two senior neuropathologists diagnosed this lesion as an NTRK fusion‐positive low‐grade glioma, not elsewhere classified (NEC).

To date, three cases of *MEF2D::NTRK1* fusion genes have been reported in CNS tumors. In 2020, a 25‐year‐old male patient with a recurrent high‐grade glioneuronal tumor in the posterior fossa harboring a *MEF2D::NTRK1* (exon 5–exon 12) fusion, along with amplification of *NTRK1* and *AKT3*, was successfully treated with 6 months of larotrectinib [12]. Infantile‐type hemispheric gliomas are typically driven by receptor tyrosine kinase (RTK) gene fusions such as *NTRK1/2/3*, *ALK*, *ROS1*, and *MET—as* 3 ^′^ fusion partners. Accordingly, a 13‐month‐old girl with a spinal lesion carrying the *MEF2D::NTRK1* (exon 6–exon 10) fusion was diagnosed with infantile hemispheric glioma [13]. After showing refractory disease following four cycles of conventional chemotherapy, she began treatment with the *NTRK* inhibitor larotrectinib and achieved a partial (< 50%) reduction within 4 months [30]. Another reported case involved a glioblastoma with a *MEF2D::NTRK1* (exon 9–exon 12) fusion, a *PIK3R1* mutation and *VEGFR2* amplification. However, detailed clinical and pathological information was unavailable [31].

All three previously reported *MEF2D::NTRK1* fusion‐positive cases were high‐grade gliomas. Among them, two patients treated with larotrectinib achieved either partial or complete remission, whereas treatment details were missing for the third patient. In contrast, we reported the first documented instance of a histologically LGG carrying a *MEF2D::NTRK1* fusion in an adult patient. A summary of the limited literature on *MEF2D::NTRK1* fusion in gliomas is provided in Table 1. Outside the glioma context, an *MEF2D::NTRK1* (exon 5–exon 12) fusion has also been reported in an 11‐year‐old patient with embryonal rhabdomyosarcoma (ERMS) [11].

**Table 1:** Reported cases of *MEF2D::NTRK1* fusion‐positive glioma in literature and the current one.

Source (author and year)	Anatomic site	Age	Diagnosis	Tumor grade	NTRK fusion breakpoint	Other molecular alterations	Targeted therapy	Progression	Follow‐up status/OS	Clinical outcome
Gianno F. et al. 2022; Di Ruscio V. et al. 2022	Spinal cord	13‐month‐old	Infant‐type hemispheric glioma	High‐grade	*MEF2D* exon 6:: *NTRK1* exon 10	Not reported (RNA‐NGS only)	Larotrectinib	Partial remission at 6 months	8 months	Alive
Tadipatri R. et al. 2022	Posterior fossa	18‐year‐old	Ganglion cell tumor	High‐grade	Not applicable	Not reported	None	Remission for 3 years, then lost to follow‐up	3 years	Alive (at last follow‐up)
	Posterior fossa	25‐year‐old	Glioneuronal tumor	High‐grade	*MEF2D::NTRK1*	*TMB1*, *NTRK1*, and *AKT3* amplifications	Larotrectinib	Remission at 6 months	19 months	Alive

Ferguson S.D. et al. 2018	Not reported	Not reported	Glioblastoma multiforme	High‐grade	*MEF2D* exon 9:: *NTRK1* exon 12	*TMB11*, *PIK3R1*, and *VEGFR2* amplification	Not reported	Not reported	Not reported	Not reported
Current case	Suprasellar cistern	33‐year‐old	Low grade Glioma, NEC	Low‐grade	*MEF2D* exon 9:: *NTRK1* exon 12	*MCL1* amplification, chromosome 5 gain and 22q loss	Entrectinib	Not progressed	19 months	Alive

Abbreviations: NEC, not elsewhere classified; OS, overall survival.

*NTRK* fusions are only observed in less than 1% of tumors overall. A broad spectrum of *NTRK* fusions has also been described in both high‐ and low‐grade CNS tumors across pediatric and adult populations [32]. Among gliomas, the majority of *NTRK* fusion‐positive cases are high‐grade. *NTRK1* fusion‐positive diffuse high‐grade gliomas (DHGGs) typically harbor more complex genetic alterations than diffuse low‐grade gliomas (DLGGs). The *NTRK1/2/3* genes can fuse with various partners in glioma, with *NTRK2* being the most frequently altered member of the *NRTK* family [19, 31]. Reported fusion partners include *TPM3*, *BCAN*, and *MEF2D* for *NTRK1*; *VCAN*, *GKAP1*, *KCTD8*, *NOS1AP*, *TBC1D2*, *SQSTM1*, and *BCR* for *NTRK2*; and *EML4* and *ETV6* for *NTRK3* [31]. In all such cases, the resulting chimeric protein retains the kinase domain of the *NTRK* gene, leading to its constitutive activation and subsequent oncogenic signaling.

For decades, the standard treatment for most adult gliomas has involved neurosurgical resection and cranial radiotherapy. Temozolomide, which is valued for its excellent CNS permeability, is the most widely used chemotherapeutic agent in adult diffuse gliomas. In particular, targeted therapies have emerged as a viable strategy for selected patients. For example, the combination of the *BRAF* inhibitor dabrafenib and the MEK inhibitor trametinib has shown clinically meaningful efficacy in patients with recurrent or refractory high‐ or low‐grade gliomas harboring the *BRAF^V600E^* mutation, with a safety profile consistent with that observed in other tumor types [33].

Since the initial FDA approval of TRK inhibitors in 2018, these agents have demonstrated remarkable activity in CNS tumors harboring *NTRK* fusions and lacking resistance mutations regardless of tumor origin, histologic subtype, or the specific *NTRK* fusion partner [7, 22]. In patients with *NTRK* fusion‐positive CNS tumors, larotrectinib is associated with rapid and durable responses, a high disease control rate, and a favorable safety profile [34]. For example, an adult patient with a malignant glioneuronal tumor bearing an *STRN1::NTRK2* fusion and wild‐type *IDH* and *BRAF* achieved a complete response to larotrectinib that lasted for 11 months, with no reported adverse events upon tumor progression [35].

TRK inhibitors exert their antitumor effects by binding to the ATP‐binding site of NTRK fusion proteins, thereby inhibiting downstream phosphorylation and blocking the transmission of proliferative signals within cancer cells. This ultimately leads to tumor regression or suppression of tumor growth [36]. The therapeutic efficacy of *NTRK*‐targeted therapy in gliomas is substantially influenced by tumor grade and the presence of co‐occurring molecular alterations. Patients with DHGGs harboring *NTRK* fusions along with other molecular abnormalities often experience disease progression despite treatment. In contrast, those with DLGGs lacking additional genetic alterations frequently achieve disease stabilization when treated with a TRK inhibitor [32]. For patients with recurrent *NTRK* fusion‐positive CNS tumors who have exhausted standard treatment options, the use of an approved *NTRK* inhibitor is recommended. However, whether patients with newly diagnosed *NTRK* fusion‐positive glioma should receive these inhibitors early either alone or in combination with standard therapy remains a subject of debate [37]. Clinical observations underscore this uncertainty: a 52‐year‐old patient with LGG harboring only a *BCR::NTRK2* fusion received standard therapy but ultimately succumbed to the disease, with an OS of 13.5 months [38]. This patient did not benefit from targeted therapy; however, the approved *NTRK* inhibitors may offer hope for future patients with similar genetic profiles.

Acquired resistance represents a major limitation of long‐term first‐generation NTRK inhibitor therapy, mainly categorized as on‐target resistance via NTRK kinase domain point mutations and off‐target resistance driven by reactivated downstream MAPK signaling [22]. To overcome such resistance, multiple second‐ and third‐generation NTRK TKIs (e.g., repotrectinib, selitrectinib, and zurletrectinib) have been developed with optimized structural affinity for mutated NTRK proteins, effectively reversing common on‐target resistance and exhibiting potent intracranial activity [39–41]. Clinically, sequential treatment with next‐generation NTRK TKIs is the recommended salvage strategy for patients with disease progression after first‐generation TKI failure [41].

In addition to the *MEF2D::NTRK1* fusion, this case also exhibited whole loss of chromosome 22q, *MCL1* amplification and chromosome 5 gain, all of which carry important biological and clinical implications. Chromosome 22q harbors multiple tumor suppressor genes (e.g., *NF2*) that regulate cell cycle stability, cell adhesion, and tumor invasiveness [42, 43]. 22q loss ablates these tumor‐suppressive pathways, accelerating tumor infiltration and disease progression [44]. Notably, concurrent 22q loss defines a high‐risk subgroup among NTRK‐fusion–positive low‐grade gliomas, conferring aggressive biological behavior and inferior clinical prognosis despite benign histology [32]. For example, consistently, non–IDH‐mutant gliomas with 22q loss exhibit markedly shorter median survival than those with intact 22q (45.3 vs. 124.7 months) [45]. The co‐occurring copy number changes further exacerbate tumor malignancy: *MCL1* amplification strengthens antiapoptotic signaling, promotes tumor cell survival, and induces therapeutic resistance to drive persistent tumor growth [46]. Whole chromosome 5 gain enhances proliferative and invasive capacities, which correlates with progressive disease in low‐grade gliomas [47]. Collectively, these genomic alterations synergize with 22q deletion to establish a high‐risk molecular profile, mechanistically interpreting the aggressive clinical phenotype of this morphologically indolent NTRK‐fusion glioma. However, clinical evidence for these next‐line agents remains limited in rare high‐risk NTRK‐fusion–positive gliomas with concurrent 22q loss, *MCL1* amplification, and chromosome 5 gain, requiring further validation in prospective cohorts.

Accurate diagnosis is essential in primary CNS tumors. Molecular alterations are becoming more essential in establishing accurate diagnosis, informing treatment strategies and providing prognostic implications. Molecular biomarkers are playing an increasingly central role in diagnostic and therapeutic decision‐making, rendering comprehensive molecular profiling indispensable for optimizing treatment selection and improving clinical outcomes.

## 4. Conclusion

This report first documented case of an adult LGG harboring a *MEF2D::NTRK1* fusion. This tumor poses a diagnostic challenge according to the WHO classification of CNS tumors, fifth edition. Histologically, it exhibited low‐grade features with mild atypia and low mitotic activity. However, molecular profiling revealed characteristics aligning with either diffuse *IDH*‐wildtype glioma or infantile‐type hemispheric glioma—both of which are classified as high‐grade. Given the presence of the *MEF2D::NTRK1* fusion, this patient underwent targeted therapy with entrectinib, which may offer clinical benefit.

**Nomenclature**
CNS: central nervous system
DLGNT: diffuse leptomeningeal glioneuronal tumor
DWI: diffusion‐weighted imaging
IDH1/2: isocitrate dehydrogenase 1/2
LGG: low‐grade glioma
LOH: loss of heterozygosity
MGMT: O‐6‐methylguanine‐DNA methyltransferase
MRI: magnetic resonance imaging
NTRK1: neurotrophic tropomyosin receptor kinase 1
OS: overall survival
PA: pilocytic astrocytoma

## Funding

This study was supported by National Natural Science Foundation of China (10.13039/501100001809; 82002721, 82203280, 82472650, 82403055) and Sichuan Province Science and Technology Support Program (10.13039/100012542; 2025ZNSFSC0552, 2025ZNSFSC1889, 2025ZNSFSC0786).

## Ethics Statement

This case was approved by Clinical Ethics Committee of West China Hospital of Sichuan University, and patient′s legal guardian gave consent, under Approval Number (2023) Ethical No. 1843.

## Consent

The patient provided written informed consent for the publication of this case report in an anonymized format and for the use of their data for research.

## Conflicts of Interest

The authors declare no conflicts of interest.

## Data Availability

The data that support the findings of this study are available from the corresponding author upon reasonable request.
